# Renal venous flow in different regions of the kidney are different and reflecting different etiologies of venous reflux disorders in septic acute kidney injury: a prospective cohort study

**DOI:** 10.1186/s40635-024-00700-0

**Published:** 2024-12-10

**Authors:** Rongping Chen, Hui Lian, Hua Zhao, Xiaoting Wang, and Critical Care Ultrasound Study Group

**Affiliations:** 1 Department of Critical Care Medicine, The First Affiliated Hospital of Sun Yat-Sen University, Guangzhou, China; 2 Peking Union Medical College Hospital, Beijing, China

**Keywords:** Renal congestion, Proximal renal venous flow, Intrarenal venous flow, Sepsis, Acute kidney injury

## Abstract

**Background:**

Acute kidney injury (AKI) is a frequent complication of sepsis. While impaired renal venous reflux indicates renal congestion, the relationship between AKI outcomes and hemodynamic parameters remains debated. This study aimed to investigate the utility of renal venous flow patterns in various regions of septic patients and to explore the association between hemodynamic parameters and renal function prognosis.

**Methods:**

In this single-center, prospective longitudinal study, adult sepsis patients diagnosed with AKI were enrolled. Renal ultrasonography was performed within 24 h of ICU admission (D1), then repeated at D3 and D5. Patterns of proximal renal venous flow (PRVF) and intrarenal venous flow (IRVF) patterns were confirmed by two blinded sonographers. Kaplan–Meier survival analysis was used to evaluate renal prognosis, and cumulative incidence curves were generated for renal function recovery time.

**Results:**

The study included 96 septic patients. Inconsistencies between PRVF and IRVF patterns occurred in 31.9%, with PRVF patterns being more severe in 88% of these. A relatively strong correlation was observed between PRVF and CVP, but this trend was less evident in IRVF. For RVSI of PRVF at ICU admission, the AUC to predict 28-day renal function prognosis was 0.626 (95% CI 0.502–0.750, *P* = 0.044), while combined PRVF and IRVF had a higher predictive ability (AUC 0.687, 95% CI 0.574–0.801, *P* = 0.003). The 28-day renal prognosis was poorer in the PRVF 5-day non-improvement group compared to the 3-day improvement group (*P* = 0.001) and 5-day improvement group (*P* = 0.012). Patients with a persistent monophasic PRVF pattern within 5 days had a worse prognosis than the non-monophasic group (*P* = 0.005).

**Conclusions:**

Our study reveals that patterns of PRVF and IRVF are not entirely congruent, stepwise evaluation is useful in determining the intervention site for renal vein reflux disorders. Combined PRVF and IRVF had a higher predictive ability for 28-day renal function prognosis. Early improvement in renal venous congestion is crucial for better renal function prognosis.

This study is registered with ClinicalTrials.gov, number NTC06159010. Retrospectively registered 28 November 2023.

## Background

Acute kidney injury (AKI) is a common complication of sepsis, occurring in up to 40–50% of hospitalized patients [1]. The recovery phenotype of AKI is strongly associated with mortality [2]. The pathophysiologic mechanisms underlying sepsis-associated AKI are multifaceted, with alterations in renal blood flow perfusion being key to its development and prognosis. While arterial supply has traditionally received more focus, recent evidence suggests that impaired venous reflux, present in up to 67% of patients, predicts adverse outcomes in critically ill individuals [3–5].

Indicators of impaired renal venous reflux, such as elevated central venous pressure (CVP) and peripheral edema, have significant limitations [6]. CVP measurement is invasive and not universally available, while peripheral edema lacks quantitative assessment and uniform standards. Intrarenal venous flow (IRVF) patterns, obtained noninvasively via Doppler ultrasonography, offer a direct method for monitoring renal venous reflux status [7, 8]. Spiegel et al. found no clear correlation between major adverse kidney events at 30 days and IRVF patterns [9]. In a small cohort of septic patients, IRVF patterns were associated with the AKI recovery phenotype but were independent of CVP [10]. Conversely, a study in heart failure patients indicated that IRVF depends on CVP and correlates with renal congestion [11]. These conflicting results hinder the clinical application of IRVF patterns.

The flow patterns in various renal veins, including intrarenal and proximal renal veins, reflect the status of renal venous reflux. Given the varying distances from the heart, IRVF can also be affected by other factors, possibly leading to inconsistencies between IRVF and proximal renal veins flow (PRVF). To date, no study has explored the utility of renal venous flow pattern in different regions among septic AKI patients. Therefore, this study aims to examine the utility of PRVF and IRVF in sepsis patients with AKI, focusing on the relationship between hemodynamic parameters and renal prognosis.

## Methods

### Study design and setting

This study was designed as a single-center prospective study, conducted at Peking Union Medical College Hospital, a tertiary hospital ranking first among all Chinese hospitals for 14 consecutive years. Serial assessments were made at 3 time points: Day 1 (D1) within 24 h after sepsis diagnosed, D1 + 48 h (D3), and D1 + 96 h (D5). The protocol received approval from the Ethics Committee of Peking Union Medical College Hospital (approval number I-23PJ176). Written informed consent was obtained from all participants. All procedures adhered to the ethical standards of the local ethics committee on human experimentation and to the Helsinki Declaration of 1975.

### Study population

This study encompassed sepsis patients diagnosed with AKI, aged 18 years or older, in accordance with KDIGO criteria between January 1, 2022, and December 30, 2023. According to KDIGO criteria, AKI stage I is defined as 1.5 to 1.9 times baseline or ≥ 0.3 mg/dl (≥ 26.5 μmol/l) increase, or urine output < 0.5 ml/kg/h for 6–12 h; AKI stage II is defined as 2.0 to 2.9 times baseline, or urine output < 0.5 ml/kg/h for ≥ 12 h; AKI stage III is defined as 3.0 times baseline or increase in serum creatinine to ≥ 4.0 mg/dl (≥ 353.6 μmol/l) or initiation of renal replacement therapy (RRT); or urine output < 0.3 ml/kg/h for ≥ 24 h or anuria for ≥ 12 h [12]. Patients with sepsis must meet the latest diagnostic criteria (Sepsis-3 defines sepsis as “life-threatening organ dysfunction caused by a dysregulated host response to infection; Sequential [Sepsis-related] Organ Failure Assessment Score (SOFA) is used to define organ dysfunction as an increase in the total SOFA score of 2 points or more). Septic shock will be classified as persisting hypotension requiring vasopressors to maintain mean arterial pressure [MAP] > 65 mmHg and having serum lactate level > 2 mmol/L despite adequate volume resuscitation [13]. Defining AKI biochemically is challenging when the baseline serum creatinine (SCr) is unknown. For patients with known baseline creatinine, AKI was identified using the lowest SCr value from the 12 months preceding admission. If no preadmission creatinine was available, the lowest value the creatinine returned to after resolution of acute illness was used. Failing all the above and if AKI could not be staged by other criteria, the admission creatinine was used as the baseline value [14]. Excluded from the study were patients who (i) exhibited chronic renal dysfunction or other systemic diseases causing renal dysfunction, (ii) had structural kidney abnormalities or disproportionate function, (iii) had ureteral obstructions affecting IRVF waveforms [15], (iv) had received a kidney transplantation, (v) were pregnant [16], (vi) were undergoing maintenance dialysis for chronic renal failure, and (vii) were unable to undergo renal ultrasound examination.

### Renal ultrasonography

**Fig. 1:**
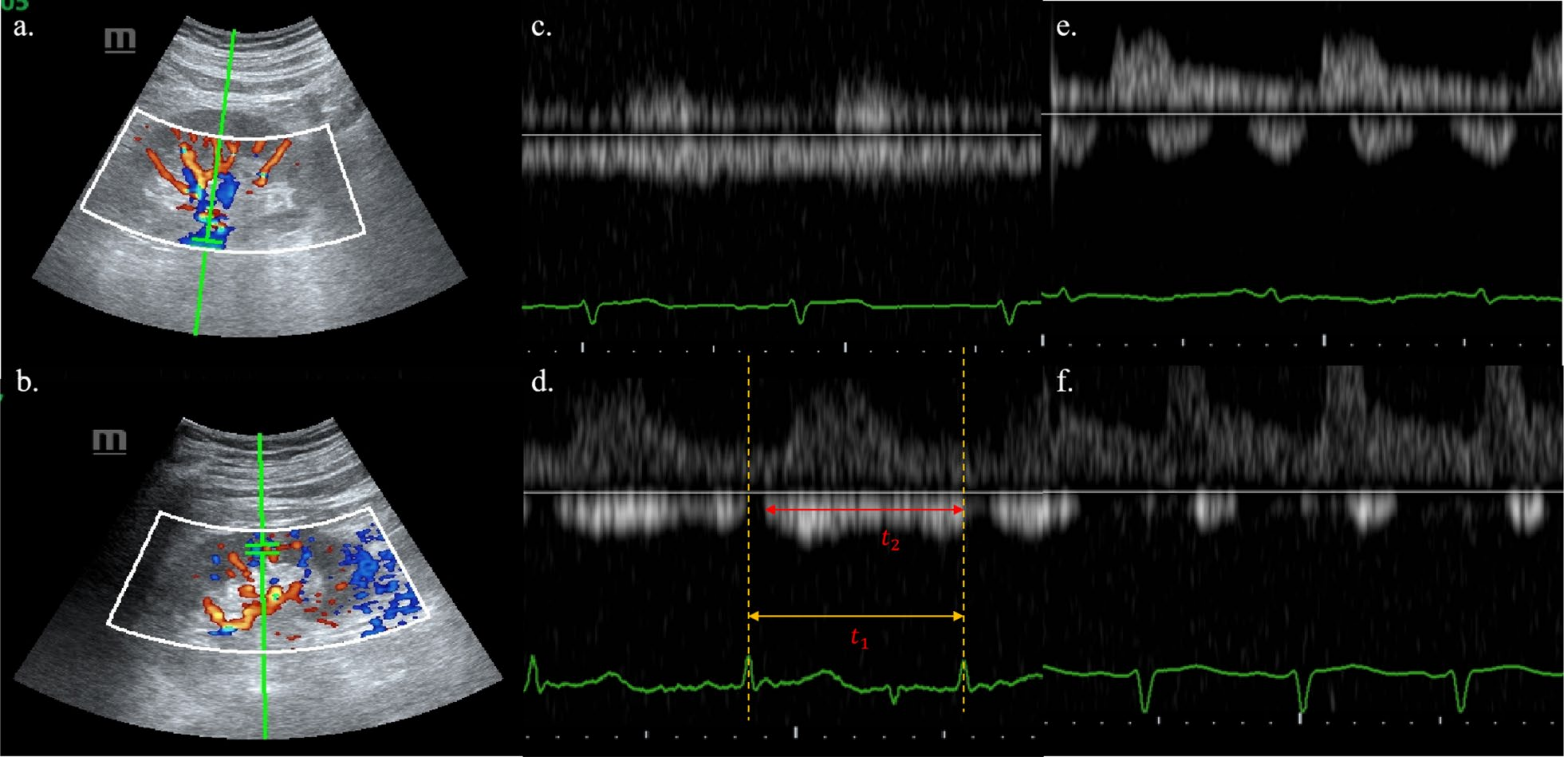
Different types of renal venous reflux status. **a** Sampling site: the proximal renal venous; **b** sampling site: the intrarenal venous; **c** continuous pattern; **d** discontinuous pulsatile pattern; **e** discontinuous biphasic pattern; **f** discontinuous monophasic pattern. The renal venous stasis index (RVSI) was calculated as the index cardiac cycle time (${t}_{1}$) minus the renal venous flow time (${t}_{2}$) divided by the index cardiac cycle time

Echocardiography was performed at the bedside in the ICU. Both kidneys were examined using a sector transducer, after which the relatively more affected side was selected for inclusion. Color Doppler images facilitated the identification of a target renal and intrarenal vein (Fig. 1). The PRVF and IRVF waveforms, representing flow away from the transducer below the baseline, were categorized into four patterns based on venous reflux status: continuous, discontinuous pulsatile, discontinuous biphasic, and discontinuous monophasic. Specifically, the continuous pattern indicated uninterrupted venous flow below the baseline throughout the cardiac cycle (Fig. 1c). In contrast, the discontinuous patterns included any pattern exhibiting at least one phase of zero velocity in venous flow during a cardiac cycle (Fig. 1d–f). The renal venous stasis index (RVSI) was employed to quantify renal reflux obstruction. This index was calculated as the index cardiac cycle time minus the renal venous flow time, divided by the index cardiac cycle time [17]. For patients in sinus rhythm, indices were measured over three cardiac cycles at the end of expiration and averaged. For patients with atrial fibrillation, measurements were taken during a cardiac cycle where the two preceding cycles had nearly equal durations [11]. PRVF, IRVF, and CVP were obtained at three time points for each patient: within 24 h after ICU admission (D1), then on D3 and D5.

Prior to this study, one investigator, proficient in ultrasound, underwent training sessions to standardize renal ultrasonography procedures. Two sonographers, blinded to all clinical information, reviewed the images and confirmed the final assessment of the PRVF and IRVF patterns. Apart from the study investigators, no other physicians were informed of the renal ultrasonography results.

### Other parameters collected

We gathered demographic information (age, gender, height, and weight) along with diagnoses. In addition, various ICU-related parameters were collected at the time of diagnosis for all patients. These included heart rate (HR), systolic blood pressure (SBP), diastolic blood pressure (DBP), mean arterial pressure (MAP), central venous-to-arterial carbon dioxide difference [P_*(V–A)*_ CO_2_], central venous oxygen saturation (ScvO_2_), Acute Physiology and Chronic Health Evaluation II score (APACHE II), and SOFA.

### Outcomes

In patients with sepsis-associated AKI, we assessed renal recovery at day 28 post-enrollment as the primary outcome. These patients were monitored for 28 days through clinical visits or telephone interviews. Nonrecovery from AKI was defined by either of the following criteria: the last creatinine measurement within the initial 28 days of hospitalization exceeding 1.5 times the baseline value, the need for RRT, or death.

### Statistical analysis

Continuous data were assessed for normality using the Shapiro–Wilk test and presented either as median and interquartile range (IQR) or mean and standard deviation (SD), based on the results. These data were then compared using the non-paired *t* test, one-way ANOVA, Kruskal–Wallis test, or Mann–Whitney U test, depending on their normality. Categorical variables were expressed as numbers and percentages and compared using the Chi-squared test. For variables not following a normal distribution, Spearman’s correlation analysis was utilized. The receiver operating characteristic (ROC) curve was used to assess the accuracy of the variables for prediction of 28-day renal function prognosis. Kaplan–Meier survival analyses were conducted to evaluate renal prognosis, and cumulative incidence curves were generated to track the time to renal function recovery. Statistical analyses were performed using SPSS software version 26.0 (IBM Corp., Armonk, NY, USA) and R software 4.2.2 (R Foundation, Vienna, Austria). All *P* values were two-sided and considered significant at < 0.05.

##  Results

**Fig. 2:**
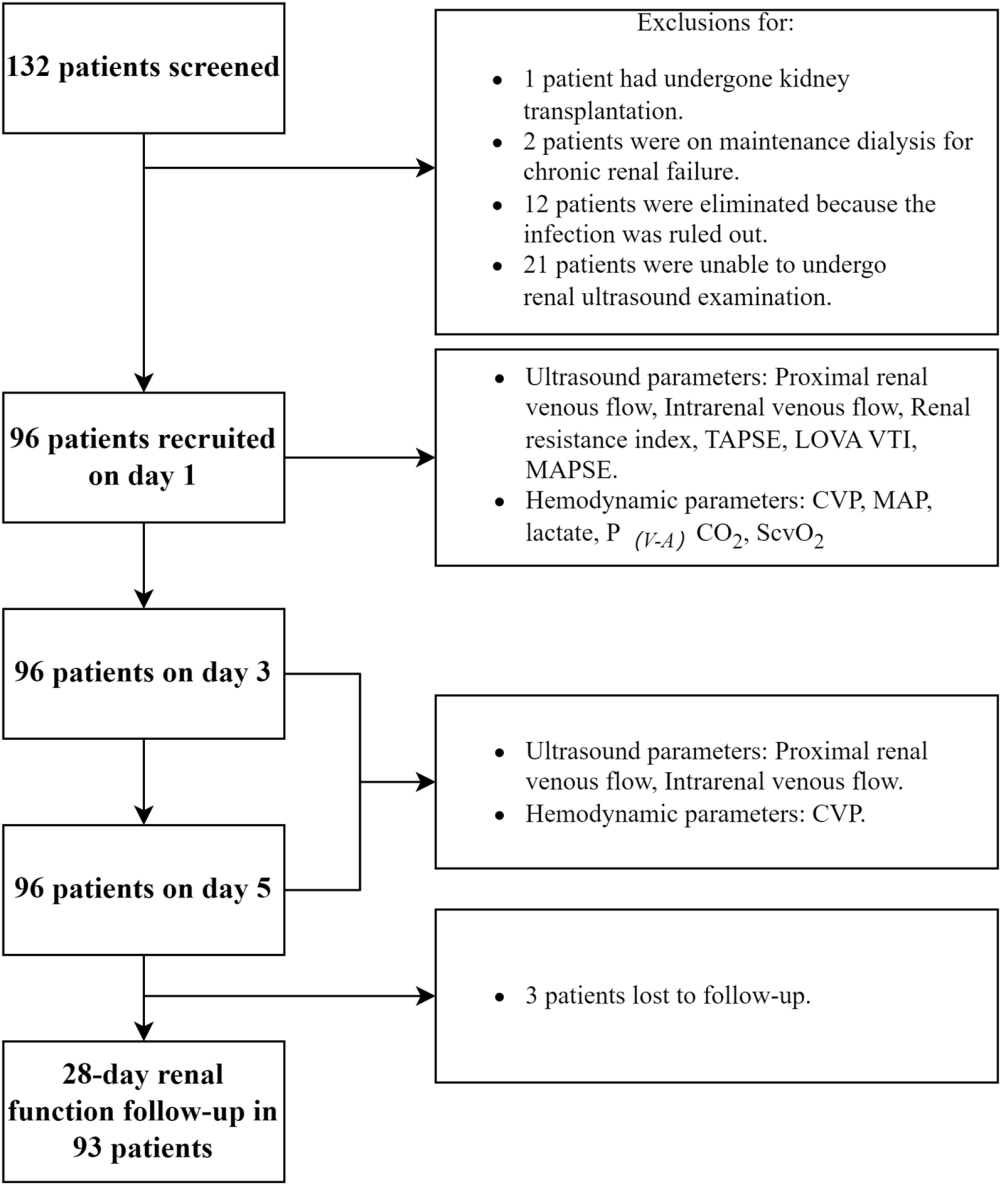
Flow diagram of participants through the study. *TAPSE* tricuspid annular plane systolic excursion, *LVOT VTI* velocity–time integral of the left ventricular outflow tract, *MAPSE* mitral annular plane systolic excursion, *P*_*(V–A)*_* CO*_*2*_ central venous-to-arterial carbon dioxide difference, *ScvO*_*2*_ central venous oxygen saturation, *CVP* central venous pressure, *MAP* mean arterial pressure

As shown in Fig. 2, 132 sepsis patients with AKI were screened during the study period. Out of these, 117 met the inclusion criteria, but 21 were excluded based on the exclusion criteria. Ultimately, 96 patients were enrolled in the study, and 93 completed the 28-day follow-up.

Table 1 presents the demographic characteristics of the study population. Among the 96 patients, 70 (72.91%) were male, with a mean age of 60.00 ± 15.37. Notably, 69 patients (71.88%) had intraabdominal infections. Most were categorized as stage one according to KDIGO criteria. In the staging of AKI, the diagnosis is based on blood creatinine level in both Stage I and Stage II, whereas in Stage III, the majority (60%) of the diagnoses are based on RRT, and the rest of the diagnoses are based on blood creatinine level. Regarding past medical history, nearly half of the participants had hypertension. For ICU-related parameters, the mean MAP was 83.96 mmHg, supported by norepinephrine. The median dose was 0.175 μg/kg/min, and the lactate level was 2.1 mmol/L upon admission. The median SOFA score was 11, the median duration of mechanical ventilation was 73 h, and the average length of ICU stay was 6 days.

**Table 1:** Demographic characteristics of the study population

N = 96	N(%) / Mean ± SD	Medium (25th percentile, 75th percentile)
Male, (%)	70 (72.91)	
Age (years)	60.00 ± 15.37	
Height (cm)		170 (165.0, 174.5)
Weight (kg)		70 (60, 80)
Infectious source, n, (%)		
Intraabdominal	69 (71.88)	
Respiratory	15 (15.63)	
Bloodstream infection	9 (9.38)	
Musculoskeletal and skin	3 (3.13)	
AKI KDIGO stage		
1	62 (64.58)	
2	14 (14.58)	
3	20 (20.83)	
Past medical history, n, (%)		
Hypertension	46 (47.92)	
Diabetes	20 (20.83)	
Cardiovascular disease	29 (30.21)	
COPD	2 (2.08)	
ICU related parameters		
HR (bpm)	87.51 ± 14.00	
SBP (mmHg)	126.10 ± 20.66	
DBP (mmHg)	65.64 ± 12.18	
MAP (mmHg)	83.96 ± 12.21	
P_*(V–A)*_ CO_2_ (mmHg)		4.6 (2.9, 6.9)
ScvO_2_ (%)		71.5 (66.0, 71.5)
Lactate (mmol/L)		2.1 (1.2, 4.4)
APACHE II score	18.96 ± 8.25	
SOFA score		11 (10, 14)
Adrenaline dose (μg/kg/min)		0 (0.00,0.01)
Norepinephrine dose (μg/kg/min)		0.175 (0.09,0.41)
PEEP (cmH_2_O)		6 (5, 8)
Time of mechanical ventilation (h)		73 (24.5, 180.5)
Length of ICU days (days)		6 (5,10)

Values are expressed as means ± standard deviation, or median (interquartile range), or number (percentage) according to normality test

*HR* heart rate, *SBP* systolic blood pressure, *DBP* diastolic blood pressure, *MAP* mean arterial pressure, *P*_*(V–A)*_* CO*_*2*_ central venous-to-arterial carbon dioxide difference, *ScvO*_*2*_ central venous oxygen saturation, *APACHE II* Acute Physiology and Chronic Health Evaluation II score, *SOFA* Sequential Organ Failure Assessment Score, *AKI* acute kidney injury, *KDIGO* Kidney Disease Improving Global Outcomes, *COPD* chronic obstructive pulmonary disease, *PEEP* positive end-expiratory pressure ventilation

**Fig. 3:**
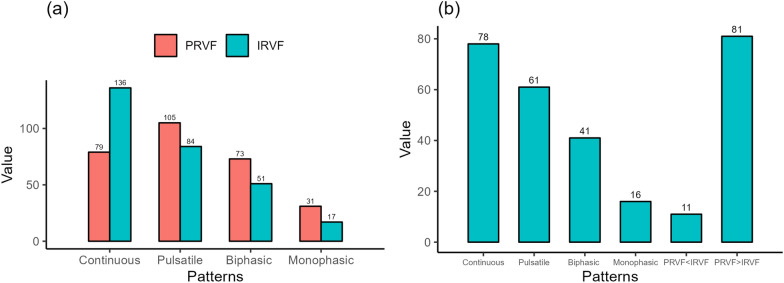
Renal venous reflux patterns of results for each examination (96 patients, 288 examinations). **a** Number of examinations in four different groups in PRVF and IRVF patterns, separately; **b** number of examinations in six different groups combined PRVF and IRVF patterns. PRVF, proximal renal venous flow; IRVF, intrarenal venous flow. PRVF < IRVF, meant obstruction of PRVF patterns were lighter than that of IRVF patterns. PRVF > IRVF, meant obstruction of PRVF patterns were heavier than that of IRVF patterns

As previously mentioned, we categorized the venous reflux flow pattern into four types: continuous, discontinuous pulsatile, discontinuous biphasic, and discontinuous monophasic. Histograms in Fig. 3a depict these four reflux types in PRVF and IRVF. In addition, Fig. 3b illustrates six combined reflux types considering both PRVF and IRVF. The PRVF patterns predominantly exhibited discontinuous pulsatile patterns (36.5%), whereas the IRVF patterns mainly showed continuous patterns (47.2%) (Fig. 3a). When comparing the consistency of the two phenotypes, the obstruction in the PRVF patterns were more severe than in the IRVF patterns (28.1%), representing a larger proportion (Fig. 3b).

Table 2 presents the hemodynamic characteristics across various PRVF and IRVF. A statistically significant difference was observed in blood lactate levels among the four PRVF (*P* = 0.040) and IRVF (*P* = 0.002) patterns. For instance, in PRVF, as venous reflux abnormalities’ severity increased among different patterns, a corresponding rise in lactate levels was noted. Among them, the lactate level in the discontinuous monophasic pattern of PRVF was significantly higher than that in the continuous pattern (*P* = 0.013), with no statistically significant differences among the other patterns. The lactate level in the discontinuous monophasic pattern of IRVF was significantly higher than that in the continuous pattern (*P* = 0.010), and the discontinuous biphasic pattern had a higher lactate level than the continuous pattern (*P* = 0.003) and the discontinuous pulsatile pattern (*P* = 0.024), with no statistically significant differences among the other patterns. The intrarenal arterial data (renal resistive index), echocardiographic parameters for both the left and right heart (TAPSE, VTI, MAPSE), pressure indicator (MAP), and perfusion indicators (P_*(V–A)*_ CO_2_, ScvO_2_) displayed similarity across the different patterns in both PRVF and IRVF. CVP and RVSI showed significant differences across the four reflux patterns, in both PRVF and IRVF (Table 2, Fig. 3). An ascending trend in both RVSI and CVP trend was observed. A statistical difference was evident in the intergroup comparison. The median CVP levels in the discontinuous monophasic pattern were recorded at 10 and 11 cmH_2_O for PRVF and IRVF, respectively.

**Table 2:** Hemodynamic characters among different types of proximal renal venous flow and intrarenal venous flow patterns

	Proximal renal venous flow patterns					Intrarenal venous flow patterns				
	Continuous (16)	Discontinuous pulsatile (23)	Discontinuous biphasic (46)	Discontinuous monophasic (11)	*P*	Continuous (34)	Discontinuous pulsatile (30)	Discontinuous biphasic (25)	Discontinuous monophasic (7)	*P*
Renal resistance index	0.65 (0.59,0.71)	0.66 (0.61,0.72)	0.66 (0.58, 0.73)	0.67 (0.64,0.79)	0.691	0.65 (0.59,0.68)	0.67 (0.61,0.73)	0.67 (0.57,0.73)	0.71 (0.62,0.78)	0.232
LVOT VTI (cm)	15.6 (14.9,17.4)	16.9 (14.8,19.3)	15.6 (14.2,20.1)	16.1 (14.4,21.1)	0.461	16.73 (15.1,21.2)	15.3 (14.3,18.4)	15.6 (14.1,18.5)	16.3 (12.8,21.0)	0.573
MAPSE (cm)	1.20 (1.1,1.5)	1.16 (1.0,1.3)	1.15 (1.1,1.3)	1.14 (1.0,1.2)	0.109	1.17 (1.1, 1.3)	1.17 (1.0, 1.4)	1.15 (1.1, 1.2)	1.12 (1.0, 1.3)	0.495
TAPSE (cm)	1.61 (1.48,1.68)	1.61 (1.43,1.78)	1.67 (1.50,1.80)	1.55 (1.43,1.67)	0.687	1.62 (1.5,1.8)	1.60 (1.45,1.67)	1.70 (1.57,1.81)	1.58 (1.46,1.67)	0.129
P_*(V–A)*_ CO_2_ (mmHg)	4.3 (1.8,6.8)	4.1 (2.9,7.2)	5.0 (3.4,6.9)	5.2 (4.3,6.6)	0.437	4.5 (1.8, 6.9)	4.1 (1.9, 6.8)	5.1 (3.8, 7.3)	5.3 (4.6, 6.5)	0.441
ScvO_2_ (%)	74.7 (66.9,77.8)	71.4 (67.8,76.3)	71.5 (68.0,75.0)	72.0 (67.4,73.5)	0.255	74.8 (69.4,77.0)	69.6 (66.0,76.9)	71.3 (68.4,73.0)	72.0 (68.0,74.0)	0.351
Lactate (mmol/L)	1.4 (1.0,1.7)* #*	2.1 (1.0,2.7)	2.2 (1.2,4.7)	3.2 (1.8,5.0)	0.040*	1.6 (1.0, 2.3)* # c*	1.8 (1.0, 2.8)* d*	3.7 (1.6, 6.4)	3.4 (2.0, 4.6)	0.002*
CVP (cmH_2_O)	6 (5,8)* #*	7 (5,9)* a*	8 (6,9)* b*	10 (9,12)	0.000*	7 (5,7.5)* # c*	7 (6,9)	8 (6,10)	11 (9,12)	0.001*
MAP (mmHg)	86.18 ± 11.77	88.22 ± 12.82	81.73 ± 11.63	89.73 ± 12.42	0.124	86.18 ± 11.79	85.10 ± 12.385	80.68 ± 12.05	80.63 ± 13.287	0.318

Values are expressed as means ± standard deviation or median (interquartile range). * Denotes significance (*P* < 0.05). *#* Denotes significance between continuous pattern and discontinuous monophasic pattern. a Denotes significance between discontinuous pulsatile pattern and discontinuous monophasic pattern. *b* Denotes significance between discontinuous biphasic pattern and discontinuous monophasic pattern. *c* Denotes significance between discontinuous biphasic pattern and continuous pattern. *d* Denotes significance between discontinuous biphasic pattern and discontinuous pulsatile pattern

LVOT VTI, velocity–time integral of the left ventricular outflow tract; MAPSE, mitral annular plane systolic excursion; TAPSE, tricuspid annular plane systolic excursion; P_*(V–A)*_ CO_2_, central venous-to-arterial carbon dioxide difference; ScvO_2_, central venous oxygen saturation; CVP, central venous pressure; MAP, mean arterial pressure

**Fig. 4:**
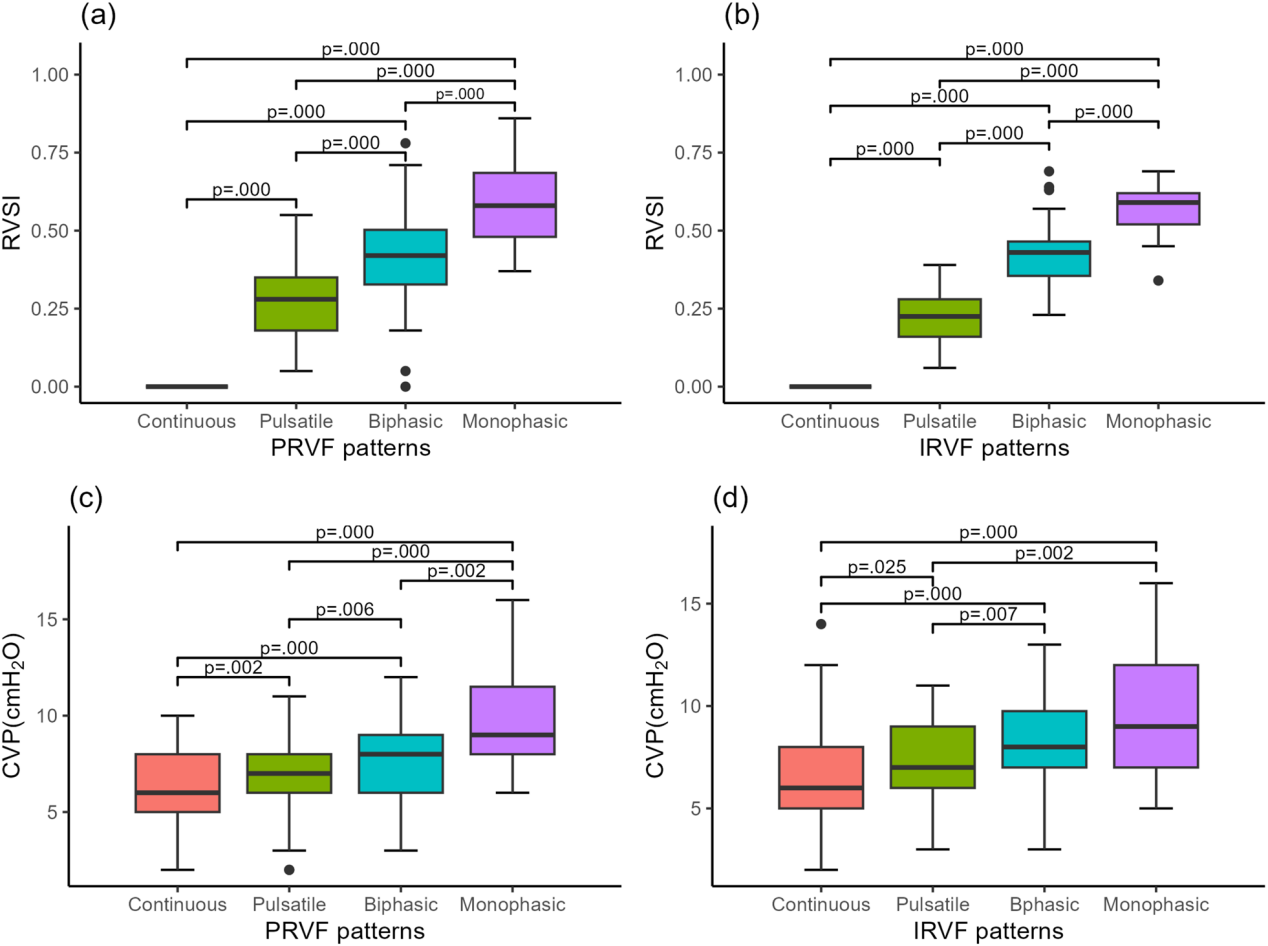
Data distribution for clinical parameters throughout the study. **a** RVSI in different PRVF patterns. **b** RVSI in different IRVF patterns. **c** CVP in different PRVF patterns. **d** CVP in different IRVF patterns. *RVSI* renal venous stasis index, *PRVF* proximal renal venous flow, *IRVF* intrarenal venous flow, *CVP* central venous pressure

Figure 4 shows the comparison of all time points data RVF patterns and IRVF patterns with RVSI, RVF patterns and IRVF patterns with CVP. RVSI of RVF in RVF patterns, the continuous pattern had a median RVSI of 0 (0,0), the discontinuous pulsatile pattern had a median RVSI of 0.16 (0,0.28), the discontinuous biphasic pattern had a median RVSI of 0.34(0.27,0.43), and the discontinuous monophasic pattern had a median RVSI of 0.58(0.44,0.68). Pairwise comparisons showed significant differences between every two patterns (Fig. 4a). RVSI of IRVF in IRVF patterns, the continuous pattern had a median RVSI of 0 (0,0), the discontinuous pulsatile pattern had a median RVSI of 0.12(0,0.31), the discontinuous biphasic pattern had a median RVSI of 0.29(0,0.46), and the discontinuous monophasic pattern had a median RVSI of 0.65(0.31,0.70). There were significant differences between every two patterns (Fig. 4b). CVP was different between the RVF patterns. The continuous pattern had a median CVP of 6 (4,8), the median CVP in the discontinuous pulsatile pattern was 7 (5,9), the discontinuous biphasic pattern had a median CVP of 7 (6,9), and the discontinuous monophasic pattern had a median CVP of 9 (7,10). Pairwise comparisons showed significant differences between the continuous pattern verse the discontinuous biphasic pattern (*P* = 0.006) and the discontinuous monophasic pattern (*P* = 0.000); the discontinuous pulsatile pattern verse the discontinuous monophasic pattern (*P* = 0.001); the discontinuous biphasic pattern verse the discontinuous monophasic pattern (*P* = 0.005) (Fig. 4c). CVP was also different between IRVF patterns. The continuous pattern had a median CVP of 6 (5,8), the discontinuous pulsatile pattern had a median CVP of 7 (5,8), the discontinuous biphasic pattern had a median CVP of 8 (6,9), and the discontinuous monophasic pattern had a median CVP of 9 (7,12). Pairwise comparisons showed significant differences between the continuous pattern verse the discontinuous biphasic pattern (*P* = 0.000) and the discontinuous monophasic pattern (*P* = 0.000); the discontinuous pulsatile pattern verse the discontinuous biphasic pattern (*P* = 0.001) and the discontinuous monophasic pattern (*P* = 0.000) (Fig. 4d).

We used the Spearman correlation coefficient to preliminarily evaluate the correlation among different groups (Table 3). A significant difference was observed between CVP and PRVF across these groups. The overall correlation coefficient was 0.498, indicating a moderately strong correlation. In the discontinuous monophasic group, this coefficient was 0.464. In contrast, the correlation with IRVF was found to be weaker. The analysis revealed no statistically significant differences in the relationship between both discontinuous biphasic and discontinuous monophasic patterns with CVP. Furthermore, we analyzed the Spearman coefficients in RVSI for the correlation between PRVF and IRVF. Here, a relatively strong correlation (*R* = 0.677) was noted. However, in the discontinuous monophasic type across different patterns, RVSI demonstrated statistical insignificance.

**Table 3:** Spearman correlations between RVSI and CVP in different groups of different patterns

Pattern	Sub-groups	CVP(cmH_2_O)	RVSI of IRVF
RVSI of PRVF	All data	**0.498**	**0.677**
	Discontinuous pulsatile	**0.325**	**0.497**
	Discontinuous biphasic	**0.432**	**0.242**
	Discontinuous monophasic	**0.464**	0.203
RVSI of IRVF	All data	**0.335**	–
	Discontinuous pulsatile	**0.222**	–
	Discontinuous biphasic	0.122	–
	Discontinuous monophasic	− 0.011	–

Bolding suggests *P* < 0.05

*RVSI* renal venous stasis index, *PRVF* proximal renal venous flow, *IRVF* intrarenal venous flow, *CVP* central venous pressure

**Fig. 5:**
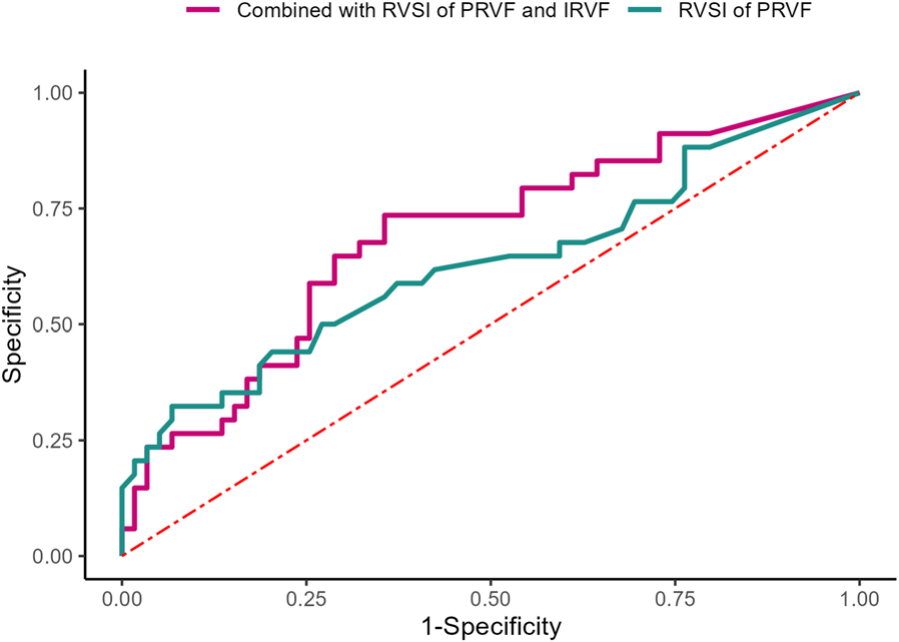
ROC for the recovery from acute kidney injury. *RVSI* renal venous stasis index, *PRVF* proximal renal venous flow, *IRVF* intrarenal venous flow pattern

For RVSI of PRVF at ICU admission, the AUC to predict 28-day renal function prognosis was 0.626 (95% CI 0.502–0.750, *P* = 0.044), with a cutoff value 0.42, sensitivity 32.4%, and specificity 93.2%. While RVSI of IRVF could not predict 28-day renal function prognosis (AUC 0.651, 95% CI 0. 497–0.806, *P* = 0.059). Combined RVSI of PRVF and IRVF had a higher predictive ability for 28-day renal function prognosis (AUC 0.687, 95% CI 0.574–0.801, *P* = 0.003), with a cutoff value of 0.43, a sensitivity of 73.5%, and a specificity of 64.4% (Figs. 5).

**Fig. 6:**
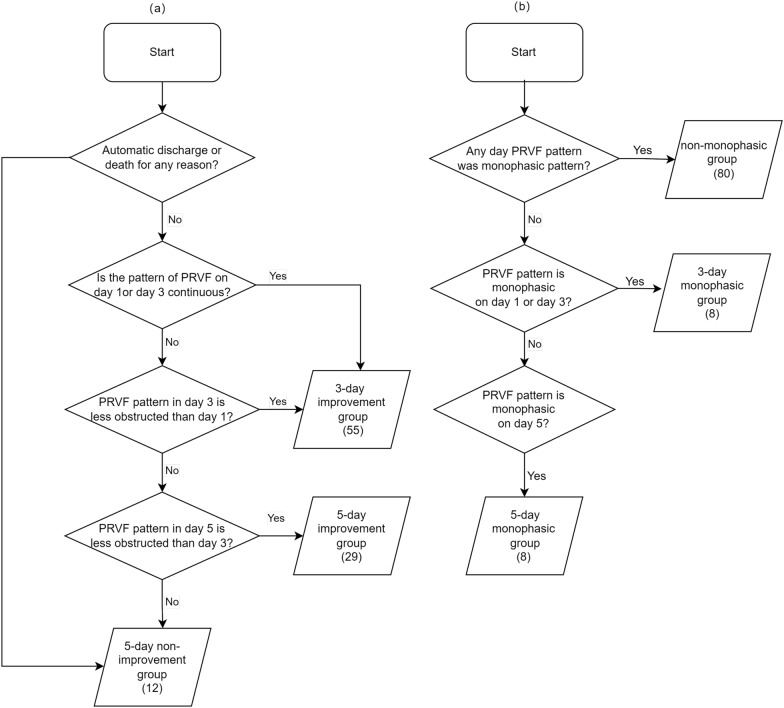
Grouping of proximal renal venous flow (PRVF) pattern is based on the flowchart. **a** Grouping of time to improvement in PRVF patterns; **b** grouping of whether the PRVF pattern is monophasic and its duration time

**Fig. 7:**
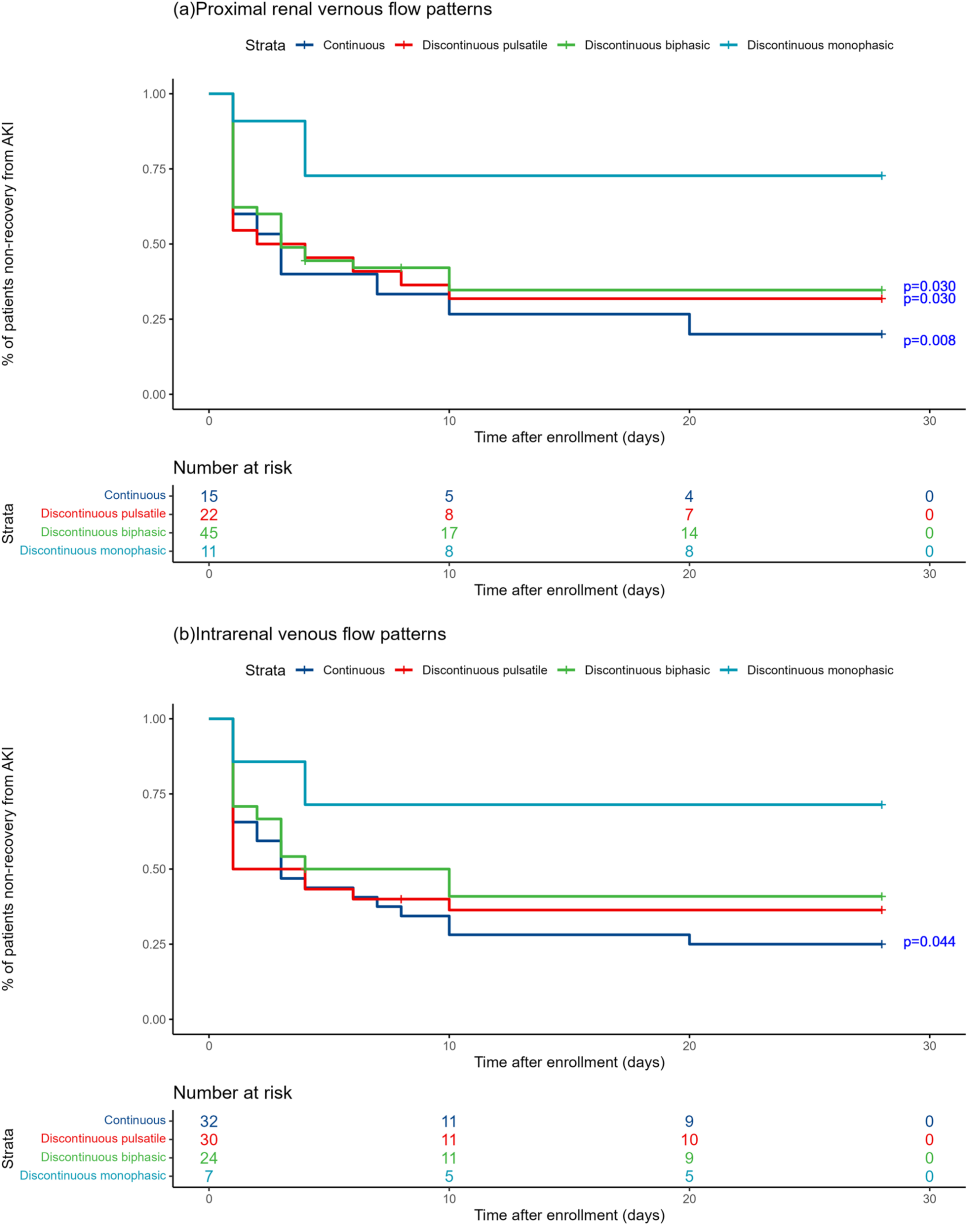

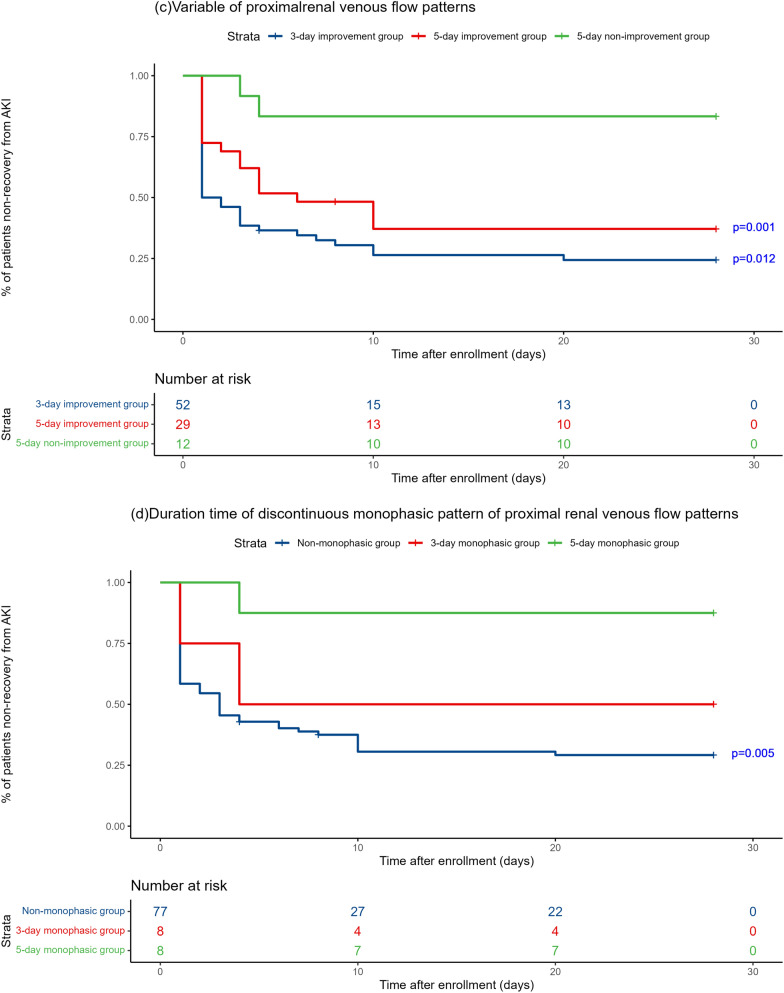
Kaplan–Meier curves for the recovery from acute kidney injury. **a, b** Non-recovery from acute kidney injury in four groups in terms of proximal renal venous flow (PRVF) pattern and intrarenal venous flow pattern (IRVF). *P* value: compared to discontinuous monophasic pattern. **c** Renal prognosis in different groups in terms of PRVF. *P* value: compared to 5-day non-improvement group. **d** Duration time of the discontinuous monophasic pattern of PRVF. *P* value: compared to 5-day monophasic group. Nonrecovery from AKI was defined as the last available creatinine during the first 28 days of hospitalization remained more than 1.5 times the baseline value, receipt of renal replacement therapy (RRT) or death

Kaplan–Meier survival estimates were used to evaluate renal prognosis (Fig. 7). Panels A and B show different groups in PRVF and IRVF patterns, respectively. In PRVF patterns on day 1, the 28-day renal function prognosis for the discontinuous monophasic group was inferior compared to that of the continuous pattern (*P* = 0.008), the discontinuous pulsatile pattern (*P* = 0.030), and the discontinuous biphasic pattern (*P* = 0.030). However, there were no statistically significant differences when comparing other groups. The trend in IRVF patterns on day 1 was not pronounced. Statistical significance was only observed between the discontinuous monophasic group and the continuous group (*P* = 0.044). Subsequent to these findings, further analysis was conducted in the PRVF pattern. Figure 6 details the methodology for evaluating changes in PRVF patterns over time (Fig. 6a) determining the presence of a discontinuous monophasic pattern at specific time points (Fig. 6b). Kaplan–Meier curve analysis revealed that the 28-day renal prognosis for the 5-day non-improvement group was poorer than that for both the 3-day improvement group (*P* = 0.001) and the 5-day improvement group (*P* = 0.012). Conversely, no significant difference was observed between the 3-day and 5-day improvement group (*P* = 0.132) (Fig. 7c). The prognosis for the 5-day monophasic group was worse compared with the non-monophasic group (*P* = 0.005). However, there were no statistically significant differences when comparing the 5-day monophasic group with the 3-day monophasic group (*P* = 0.100) or the 3-day monophasic group with the non-monophasic group (*P* = 0.268) (Fig. 7d). In summary, transitioning from a monophasic pattern to any other pattern significantly improves renal prognosis, which should be considered a therapeutic target in clinical practice.

## Discussion

To the best of our knowledge, this is the first study to investigate various venous reflux patterns and their role in assessing renal prognosis in both PRVF and IRVF. Our research team demonstrated that in sepsis-associated AKI, PRVF may be more sensitive than IRVF for renal prognosis assessment and combining PRVF and IRVF can improve the prediction of renal prognosis. Variations in venous flow patterns, the resolution of obstructed patterns, and the duration of the discontinuous monophasic pattern significantly influenced the 28-day renal prognosis. In PRVF, all subtypes of discontinuous patterns were associated with CVP, while in IRVF, the RVSI was positively correlated with CVP exclusively in the discontinuous pulsatile pattern.

The patterns of PRVF and IRVF were not entirely identical in the study population. Under physiological conditions, renal veins maintain a continuous flow independent of renal function [18, 19]. As venous load increases, cardiac pressure waves propagate further, leading to alterations in the venous blood flow spectrum. Consequently, RVF patterns vary with the severity of venous reflux disorder [20]. The inconsistency between PRVF and IRVF patterns was observed in 31.9% of cases, with the PRVF pattern being more severe in 88% of these instances. Several factors might explain these observations: Firstly, distance plays a role. The proximity of PRVF to the heart often results in a pulsatile flow, whereas IRVF demonstrates a less severe pattern owing to the “ripple” effect. If renal venous reflux impairment is solely as a result of increased right heart pressure, this typically results in either a consistent pattern or PRVF being more severe than IRVF [21]. Second, the possibility of false negatives should be considered. When renal interstitial pressure significantly increases for various reasons, leading to a marked reduction in intrarenal venous compliance, IRVF often fails to respond to downstream pressure changes. This results in a persistent flat flow without pulsatility [21–23], which might be incorrectly classified as continuous, masking a significantly impaired reflux state. Our study also identified a minority of cases where IRVF patterns were more severe than those of PRVF. This discrepancy could be attributed to renal interstitial edema without hydrostatic pressure increase, which is often caused by the destruction of renal endothelial glycocalyx and increased endothelial permeability due to factors like sepsis, inflammation, stress, and trauma [24]. Therefore, a persistent flat flow in IRVF does not necessarily imply optimal renal function. This could explain the weak correlation between IRVF and PRVF, particularly in cases of severe obstruction. As PRVF and IRVF are influenced by different factors, a stepwise evaluation of venous reflux status across various renal regions is crucial for determining the optimal site for hemodynamic intervention.

Elevation in CVP impacts venous reflux as it reflects the pressure within the right heart, the endpoint for venous reflux. Therefore, we compared the RVSI with CVP in patients with PRVF and IRVF, respectively. The results of the study indicated distinct patterns between PRVF and IRVF, with their relationship to CVP also varying. In PRVF, RVSI exhibited a significant correlation with CVP across all reflux patterns. However, in IRVF, a significant correlation was observed only in the discontinuous pulsatile pattern. Previous studies primarily targeting IRVF did not reach the same conclusions. The direct connection of the proximal renal vein to the inferior vena cava facilitates easier pressure transmission [21], with its flow pattern largely influenced by downstream pressure (indicated by CVP) and changes in vascular compliance. Moreover, the intrarenal vein’s function is altered by additional factors, such as interstitial edema from enhanced endothelial permeability [25], sensitivity to sympathetic nervous system activation, and the risk of false negatives [26–29]. This can explain why earlier studies on IRVF found associations between cardiac dysfunction patients and CVP (attributable to increased downstream pressure) [11], whereas sepsis patients showed no CVP association (due to concurrent increased endothelial permeability and downstream pressure) [10]. Based on the above information, our research results suggest that high CVP may lead to renal congestion, with a positive linear relationship between them. Under certain conditions, the higher the CVP, the more severe the renal venous flow congestion assessed by ultrasonography. However, renal congestion is not only influenced by downstream pressure but also by local vascular elasticity. In septic or septic shock states, due to widespread endothelial leakage, interstitial pressure increases, leading to decreased local vascular elasticity and aggravated venous congestion. This means that in clinical practice, excessive resuscitation leading to high CVP should be avoided, as it serves as the downstream pressure for renal venous reflux and is closely related to renal congestion. Avoiding high CVP is beneficial to reducing renal congestion which is beneficial for improving adverse renal outcomes. Our study further demonstrated that the severity of obstruction escalation correlated with lactate levels. According to Guyton’s theory of venous reflux, increased right atrial pressure reduces venous return, leading to systemic venous congestion and impaired lactate clearance [30, 31]. Although few studies focused on the correlation between RVSI and lactate levels, evidence supporting a positive correlation between CVP, as a surrogate for RVSI, and lactate levels exists [32]. Higher CVP was associated with diminished lactate clearance [33].

In terms of renal prognosis, our research demonstrated that in sepsis-associated AKI, PRVF may be more sensitive than IRVF for renal prognosis assessment and combining PRVF and IRVF can improve the prediction of renal prognosis. The discontinuous monophasic pattern exhibited poorer outcomes compared with other patterns in PRVF. A similar pattern was observed in IRVF. Recent studies have focused on the prognostic assessment of renal function in IRVF. A study involving septic patients demonstrated that analyzing IRVF patterns after 24 h correlated with the future prognosis of renal function [10]. RVSI can independently predict morbidity and mortality in individuals with pulmonary hypertension [17]. In the context of ICU patients, monitoring IRVF within the first 24 h did not correlate with renal prognosis, attributed to a variety of factors [9]. Elevated CVP is linked to the discontinuous phenotypes of renal reflux mentioned previously. Concurrently, the sympathetic nervous system (SNS) and the renin–angiotensin–aldosterone system are affected by renal venous congestion [29]. Venous tone, regulated by the SNS, is a critical determinant of effective circulatory volume [26]. SNS activation further leads to renal vasoconstriction and a decline in glomerular filtration rate [28, 34]. In addition, there is increasing interest in the roles of inflammation and endothelial cell activation [24]. These diverse mechanisms contribute to the potential irreversibility of impaired renal function.

In the context of PRVF phenotypes and their correlation with renal prognosis, we investigated the correlation between varying temporal patterns and renal prognosis in PRVF. Our study found that an inability to alleviate obstruction beyond 5 days poses a risk for chronic renal injury. Moreover, the persistence of a discontinuous monophasic pattern by day 5 is associated with a poorer renal prognosis. Immediate adjustment of venous reflux is beneficial. This finding aligns with prior research [35]. Based on our definition of adverse renal outcomes, and by examining the relationship between renal venous flow patterns and adverse renal outcomes, we found that when the renal venous flow exhibits a monophasic pattern, early AKI is more severe, with higher AKI stages, longer AKI recovery times, and a higher incidence of adverse renal outcomes. Conversely, when the pattern is non-monophasic, AKI stages are lower, indicating less AKI, shorter AKI recovery times, and a lower probability of adverse renal outcomes. Furthermore, early improvement in renal venous congestion facilitates early recovery from AKI and reduces the risk of adverse outcomes. However, we aim to highlight that any hemodynamic modification enhancing venous reflux is beneficial for renal outcomes. Minimally, transitioning patients away from the discontinuous monophasic type is crucial. Both PRVF and IRVF hold significant clinical relevance throughout the treatment process. Our results suggest considering PRVF as an indicator of systemic reflux and IRVF as reflective of organ-specific reflux. Even when systemic reflux normalizes, organ function may remain compromised. In addition to normal systemic reflux, it is critical to address organ-specific reflux. Hemodynamic interventions should be tailored accordingly. Enhancing both systemic and organ-specific reflux conditions is essential for the recovery of organ function.

### Advantages and limitations

Our study offers several advantages. First, it establishes the distinct differences between PRVF and IRVF in the kidney, with PRVF demonstrating a more pronounced degree of obstruction than IRVF. Furthermore, PRVF is closely associated with CVP, indicating that the severity of reflux obstruction correlates with the strength of this relationship. Second, our study illustrated that PRVF may be more sensitive than IRVF for renal prognosis assessment and combining PRVF and IRVF can improve the prediction of renal prognosis. We identified that phenotypes of PRVF and IRVF, the timeline for obstruction improvement in PRVF pattern, the discontinuous monophasic pattern, and its duration are significant factors affecting 28-day renal function prognosis. Third, the prognosis for 28-day renal function in cases of PRVF obstruction without improvement for over 5 days was less favorable compared to cases that showed improvement within 3 and 5 days, and outcomes were worse for those with a discontinuous monophasic pattern for 5 days than for the non-monophasic group. These findings offer new insights for intensivists and provide valuable guidance for clinical practice.

However, this study also has some limitations. First, it is a single-center study with a small sample size, and the subgroup with a discontinuous monophasic pattern is particularly limited. While our findings are promising, they require confirmation through further research with a larger cohort. Second, some of the results remain inadequately explained, indicating a need for more comprehensive studies in the future. Third, the absence of long-term follow-up might lead to an underestimation of renal function, suggesting the necessity for extended observation periods in subsequent studies. Finally, renal ultrasonography was performed by a single sonographer who were aware of the patients’ clinical information, which may lead to a high degree of subjectivity in the obtained ultrasound images and result in certain biases. However, we attempted to minimize bias from awareness of clinical information by adopting an assessment of the RVF patterns by two independent expert sonographers who were blind to the patients’ clinical data.

## Conclusions

Our study revealed that the patterns observed in PRVF and IRVF were not entirely identical. Therefore, a stepwise evaluation of venous reflux status across different regions of the kidney is beneficial for identifying the optimal site for hemodynamic intervention. Patients presenting with a discontinuous monophasic PRVF pattern at admission exhibited a poorer renal prognosis. Combining PRVF and IRVF can improve the prediction of renal prognosis. Early improvement in the PRVF pattern is associated with better renal function prognosis. It is crucial to transition patients away from the discontinuous monophasic PRVF pattern.

## Data Availability

The dataset used and analyzed for the current study is available from the corresponding author upon reasonable request.

## Abbreviations

AKI: Acute kidney injury
ANOVA: Analysis of variance
APACHE II score: Acute physiology and chronic health evaluation II score
CKD: Chronic kidney disease
COPD: Chronic obstructive pulmonary disease
CVP: Central venous pressure
DBP: Diastolic blood pressure
ICU: Intensive care unit
HR: Heart rate
IRVF: Intrarenal venous flow
KDIGO: Kidney disease improving global outcomes
LVOT VTI: Velocity–time integral of the left ventricular outflow tract
MAP: Mean arterial pressure
MAPSE: Mitral annular plane systolic excursion
MIMIC-III: Multiparameter intelligent monitoring in intensive care
NE: Norepinephrine
PEEP: Positive end-expiratory pressure
P_*(*__*V–A*__*)*_ CO_2_: Central venous-to-arterial carbon dioxide difference
RAP: Right atrial pressure
PRVF: Proximal renal venous flow
RRT: Renal replacement therapy
SCr: Serum creatinine
ScvO_2_: Central venous oxygen saturation
SD: Standard deviation
SNS: Sympathetic nervous system
SOFA: Sequential organ failure assessment
TAPSE: Tricuspid annular plane systolic excursion
VII: Venous impedance index
